# Study on the impact of China’s urban agglomerations’ tiered spatial structure on regional economic resilience

**DOI:** 10.1371/journal.pone.0314538

**Published:** 2025-03-13

**Authors:** Wei Liang, Deqi Wang, Luqin Gao, Chunyan Li

**Affiliations:** 1 School of Urban Economics and Public Administration, Capital University of Economics and Business, Beijing, China; 2 School of Management, Wenzhou Business College, Wenzhou, China; Wuhan University, CHINA

## Abstract

Urban agglomerations serve as crucial spatial carriers of economic development, and their spatial structure profoundly influences regional economic resilience. This study draws on Martin’s conceptualization of economic resilience and, considering the administrative hierarchy and development stage of China’s urban system, examines the impact of the layered spatial structure of urban agglomerations on regional economic resilience. Based on data from 17 Chinese urban agglomerations from 2005 to 2019, this research employs a one-step system Generalized Method of Moments (GMM) model to empirically analyze the effects of three mechanisms – polycentricity within urban agglomerations, inter-city development disparities, and inter-city industrial gradients – on regional economic resilience. The findings reveal that both the polycentric distribution of population and economy and the coupling of dual centers significantly positively affect the economic resilience of urban agglomerations. The potential development energy difference between core and peripheral cities can be transformed into developmental momentum for peripheral cities, thereby generating positive spatial externalities that play a significant and positive role in enhancing economic resilience. As the ratio of secondary to tertiary industry employees in peripheral and core cities nears 1, urban agglomerations’ economic resilience strengthens, underscoring the importance of a balanced industrial gradient and regional collaboration in mitigating economic shocks. This study also considers the heterogeneity of whether the urban agglomerations are coastal and whether they contain a national-level central city. The research finds that for inland urban agglomerations and those with existing national-level central cities, developing a polycentric spatial structure can effectively enhance the region’s economic resilience in responding to various shocks. Furthermore, aside from a few more developed coastal urban agglomerations, other inland urban agglomerations should continue to focus on spatial agglomeration, fostering core cities to strengthen economic competitiveness. Finally, given the varying industrialization stages and structures within urban agglomerations, appropriately adjusting the industrial gradient is essential.

## Introduction

Regional economic development is shaped by various disturbances, including government policies, economic cycles, and technological changes. Regions display varying capacities in responding to economic shocks and recovering post-crisis. For example, the COVID-19 pandemic caused GDP growth rates in Beijing, Shanghai, and Shenzhen, China, to drop from 6.1%, 6%, and 6.7% in 2019 to 0.7%, –0.2%, and 3.3% in 2022, respectively. The reasons for differing development speeds are complex. Economic geographers use the concept of regional economic resilience to explain the varying performances of regions in resisting and recovering from shocks.

The concept of resilience was originally widely used in physics, engineering, and ecology to describe a system’s ability to maintain stability and revert to its original state after external shocks. Inspired by this concept, economists began to integrate ideas from other disciplines, infusing economic connotations into the notion of resilience. In 2002, Reggiani introduced resilience into spatial economics, applying it to examine how regional economic systems respond to shocks, disturbances, and recover afterward [1]. From an evolutionary perspective, Simmie and Martin (2010) argued that the ongoing adaptation of regional economic components to complex changes is crucial in economic resilience research [2]. Since then, resilience has been extensively applied in economic geography, with growing interest in economic and regional economic resilience [3,4]. However, at this stage, research had not yet developed a systematic definition or theoretical framework [5]. The conceptual boundaries and theoretical connotations remained vague, and quantitative analysis based on economic theory was lacking. Martin (2012) systematically reviewed cross-disciplinary literature, integrating mainstream economic theories to consolidate and extend the concept and connotations of economic resilience [6]. He developed a regional economic resilience framework with four dimensions: resistance (sensitivity), recovery (speed and extent), re-orientation, and renewal, establishing the theoretical foundation for future research. With the advent of quantitative analysis and econometric paradigms, many scholars measured regional economic resilience and examined its influencing factors from multiple perspectives. Martin et al. (2016) found that economic resilience evolves over time and that different cities exhibit significantly varied responses to economic recession shocks [7]. Many scholars have used employment data to study the impact of economic structure on regional economic resilience. They found that regions with industrial diversification and advanced industry specialization responded better to external shocks [8]. Service sector-concentrated areas demonstrated stronger resilience compared to manufacturing agglomerations, whereas regions heavily reliant on a single industry showed weaker economic resilience [9]. Chinese scholars, using GDP growth rate variations, found that fixed asset investment, manufacturing agglomeration, and government spending enhanced regional economies’ resistance to external shocks, while service sector agglomeration was crucial for economic recovery [10]. Giannakis (2017) found that accessibility, education levels, and economic development positively impact regional resilience in his study on the varied responses of European regions to economic crises [11]. Gillian (2017) found that innovation can drive regional “evolution,” thereby enhancing economic resilience [12]. Faggian (2018) found that regions with larger populations and proximity to cities possess stronger abilities to withstand shocks. Additionally, regions focused on the food, textile, and tourism industries exhibited greater resilience to shocks [13].

Beyond economic and social factors, the spatial structure of cities significantly contributes to differences in economic resilience. Green (2007) defined polycentric spatial structure from a functional perspective, suggesting that the functional connections among various entities within a city form the foundation of polycentricity [14]. Hall P. (2009), using the “Polycentric Urban Region” in Europe as an example, found that a polycentric urban development model enhances regional competitiveness and promotes coordinated development within the region [15]. Wei et al. (2022) studied the impact of monocentric structures on urban employment growth and found a U-shaped relationship, where increased compactness enhanced employment growth [16]. As urban functions and regional forms evolve, city agglomerations have emerged as a more advanced spatial organization, prompting scholars to focus on regional economic resilience within these agglomerations. Zhang et al. (2021), in their study of the Yangtze River Delta’s urban spatial network, found that the network is evolving toward a polycentric form, with inter-city connections and resilience gradually strengthening [17]. Other studies have shown that the degree of polycentricity in urban agglomerations’ spatial structure exhibits a “U-shaped” relationship with the urban–rural income gap in the region [18,19]. Lu et al. (2022) argued that cities within an urban cluster form a regional economic network, and this tightly connected structure significantly enhances economic resilience, with polycentric spatial structure acting as a driving force [20]. However, some scholars, from a sustainable development perspective, argue that the polycentric economic development model increases environmental and ecological burdens, initially promoting but eventually inhibiting green development efficiency, and contributing to air pollution [21,22].

In summary, academic research on regional economic resilience has mainly focused on three areas: defining and clarifying its conceptual boundaries and theoretical connotations; measuring and evaluating differences in economic resilience within specific regions through case studies; and exploring various factors and mechanisms influencing regional economic resilience through empirical research. Although there is extensive research on the factors influencing regional economic resilience, few studies have explored how China’s administrative hierarchy and urban cluster development characteristics impact spatial structures and, consequently, regional economic resilience. Due to the relatively late development of China’s urban system, the immature state of urban cluster development, and the strong influence of government administrative hierarchy, the spatial form of Chinese urban agglomerations falls between metropolitan areas and mature urban agglomerations. This spatial structure, centered around one or a few large cities with high administrative levels and surrounded by numerous small- and medium-sized cities, can mitigate the challenges faced by mega-core cities by decentralizing certain functions to peripheral sub-tier cities. Additionally, it can foster the development of peripheral cities through mechanisms such as scale borrowing, the establishment and sharing of infrastructure and public services, and industrial linkages. Therefore, this paper aims to elucidate the evolutionary mechanisms of the layered spatial structure of urban agglomerations under the influence of China’s administrative hierarchy and to explore its impact on regional economic resilience. The marginal contributions of this paper are as follows: First, it empirically analyzes the effects of population and economic polycentricity, development gaps between “core-periphery” cities, and industrial gradients on regional economic resilience, based on the connotation of the layered spatial structure of Chinese urban agglomerations. This effectively supplements existing research on the factors influencing regional economic resilience and holds significant theoretical implications. Second, considering the varying geographical locations, developmental stages, and city sizes within China’s 19 urban agglomerations, this paper further examines the heterogeneity in the impact of the layered spatial structure of urban agglomerations on regional economic resilience, which has practical significance.

The paper is structured as follows: Chapter 2 elaborates on the theoretical connotation of the layered spatial structure, explains its mechanisms of influence on regional economic resilience, and formulates research hypotheses. Chapter 3 outlines the empirical research design, constructs the econometric model, and introduces the variables and data sources. Chapter 4 presents the empirical research, encompassing the main regression tests, model robustness tests, and heterogeneity tests, followed by an analysis of the results. Chapter 5 summarizes the research findings, draws conclusions, and provides recommendations and discussion.

## Theoretical and hypotheses

### The concept and connotation of regional economic resilience

Resilience originally emerged as a concept in engineering, describing the ability of a material or system to maintain stability and return to equilibrium after external forces are applied. It was later extended to the domains of ecological and evolutionary resilience [23]. Martin (2012) provided a precise definition of regional economic resilience as the ability of an economic system to resist risks during a shock, recover to an equilibrium state afterward, and adapt to new environments by shifting development pathways through structural adjustments and evolutionary capacity [6]. The shock resistance and recovery capabilities within regional economic resilience align more closely with engineering resilience, while adaptability and evolutionary capacities are more akin to ecological and evolutionary resilience.

A city’s spatial structure is typically shaped by factors like its level of economic development, population agglomeration scale, and industrial location. When external shocks occur, the city’s level of economic and social development acts as an “immune system” to resist these shocks, while its economic structure, production pathways, and policy regulation serve as recovery mechanisms aiding in “post-illness” recuperation. In a larger region, the industrial networks between cities, development gaps, flows of population and resources, and the degree of integrated development constantly evolve, profoundly influencing the region’s spatial structure. Unlike city–level economic resilience, regional economic resilience in urban agglomerations and metropolitan areas places greater emphasis on the impact of internal spatial linkages. Generally, urban agglomerations with close economic ties among member cities have a larger economic scale and stronger “immunity” against economic disturbances. Urban agglomerations where “leading” core cities maintain a certain development gap with peripheral cities and possess a reasonable industrial gradient can more rapidly adapt to post–shock environments and better adjust to a new equilibrium.

### Evolutionary mechanism of urban agglomerations’ tiered spatial structure

**Fig 1:**
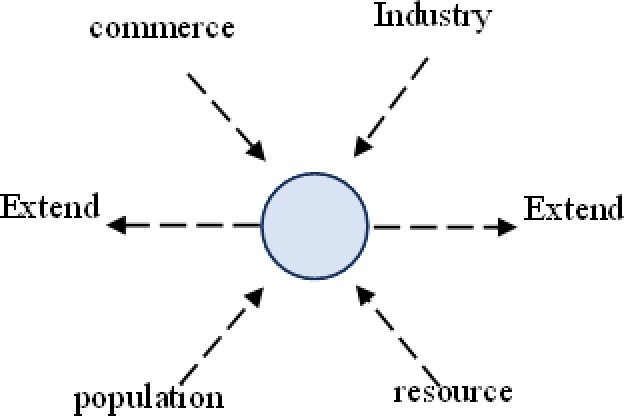
Schematic diagram of “dot-like” structures.

**Fig 2:**
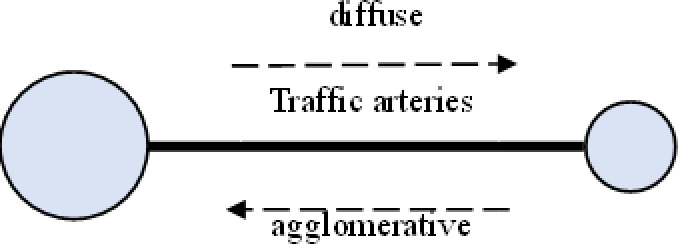
Schematic diagram of “axial” structures.

**Fig 3:**
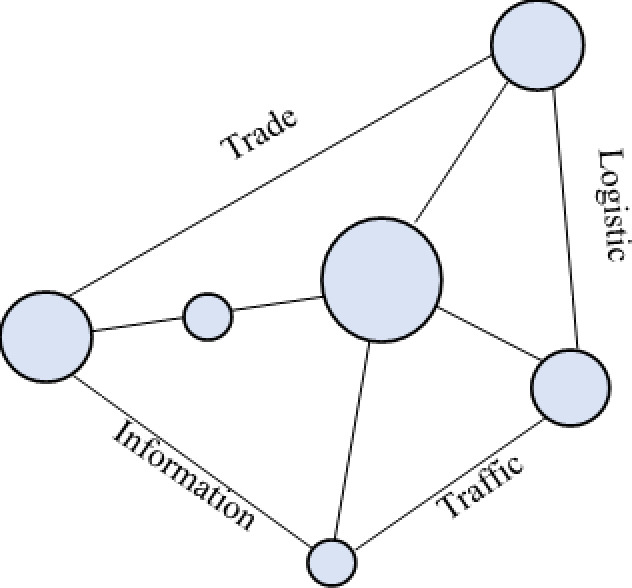
Schematic diagram of “network” structures.

**Fig 4:**
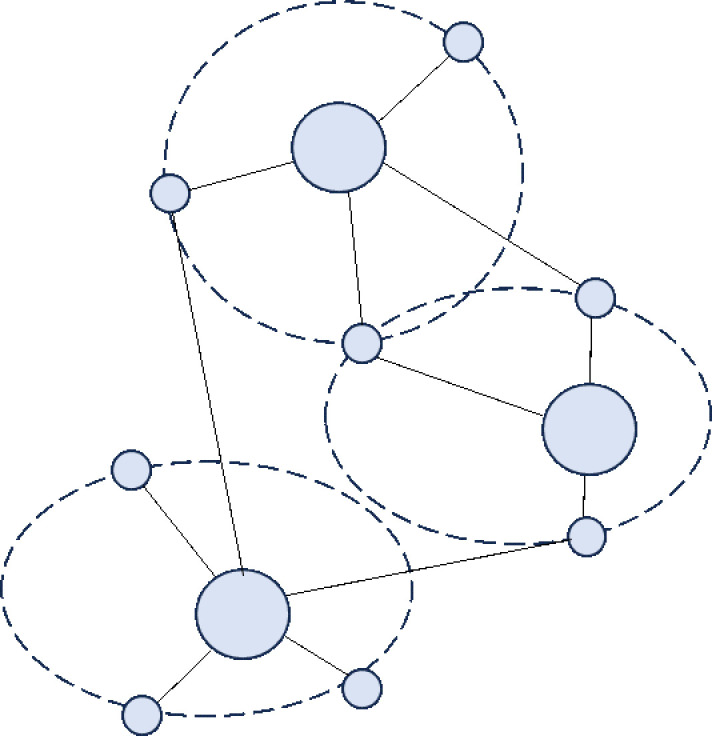
Schematic diagram of “planar” structures.

Urban agglomerations, surpassing cities in spatial organization, offer greater support for populations and economies, along with broader functionalities. Their spatial structures evolve alongside the growth of cities and diversification of economic activities. The spatial structure of urban agglomerations in developed countries typically progresses through four stages: “point–axis–network–surface (belt).” Initially, cities were independent city–states with few connections, resulting in a “point-like” distribution across vast regions (Fig 1). Following the Industrial Revolution, increased industrial productivity and resource demands led to rapid urban population concentration. Benefiting from advancements in transportation infrastructure, cities expanded along transportation arteries, closely connecting with neighboring cities via these arteries, forming “axis-shaped” urban areas (Fig 2). During this period, cities grew in population and economic size, becoming regional central cities. The refinement of industrial networks and labor division led regional central cities to form competitive-cooperative relationships with surrounding cities, creating urban agglomerations with distinct hierarchies centered around mega-cities. This resulted in “network-shaped” metropolitan areas or localized urban agglomerations, geographically close and economically and socially interconnected (Fig 3). Entering the later stages of industrialization and urbanization, interactions between cities became more orderly, and economic and social development became highly integrated spatially. Metropolitan areas and localized urban agglomerations, relying on relatively flat geographic conditions and modern transportation and internet facilities, expanded outward in an organized manner, forming continuous “surface (belt)-shaped” urban complexes with surrounding areas. (Fig 4). This process culminates in the creation of large urban agglomerations or metropolitan belts.

**Fig 5:**
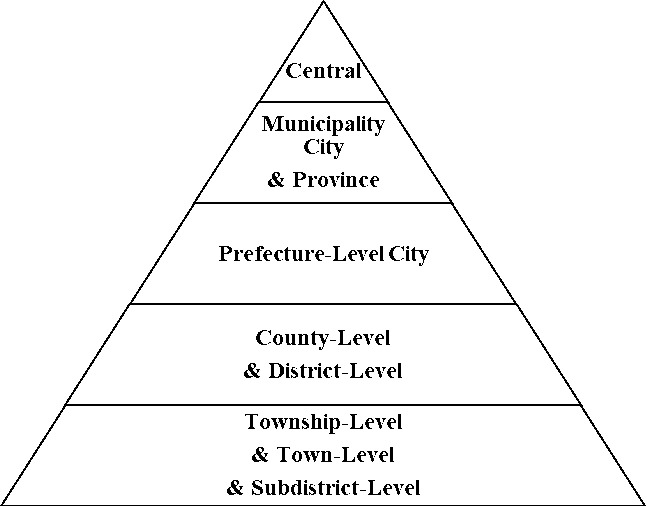
China’s five-level administrative management system.

**Fig 6:**
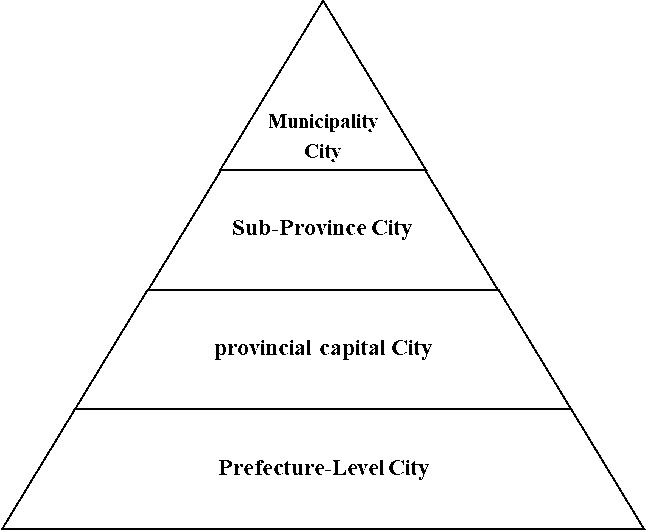
The hierarchical system of Chinese cities.

As the largest emerging economy, China’s urban agglomeration development generally adheres to the described principles. However, the development of China’s urban agglomerations exhibits characteristics unique to the country’s national conditions. China’s urban agglomerations are marked by a low beginning, delayed start, and swift development. At the founding of the People’s Republic of China, the urbanization rate was only about 10.64%. During this period, China had a weak industrial base, simple economic structure, and outdated transport facilities, with cities spread as independent “points” across vast areas. In the 20 years post-reform and opening-up, urbanization grew by an average of just 0.766% annually, with only 22 cities housing over 2 million non-agricultural residents. The development of cities and their agglomerations accelerated at the start of the 21st century, spurred by a large–scale city–building movement and infrastructure development. By 2020, the urbanization rate hit 63.89%, a 26.23% increase over 20 years, averaging 1.38% annual growth. The count of cities with over 2 million non–agricultural residents rose to 112, supporting 19 urban agglomerations at different development stages and with various scales and advantages. Additionally, China’s urban system is significantly shaped by its administrative hierarchy. The national administrative management system is structured from top to bottom into five levels of government: “Central–Provincial (Municipality City)–City–County (District)–Township (Town)” (Fig 5), implementing a strict bureaucratic management. In China’s urban system, a city’s administrative level and the rank of its chief administrative officer determine the city’s hierarchy (Fig 6), ranging from municipalities directly under the central government, sub–provincial cities, ordinary provincial capital cities to ordinary prefecture–level cities. Among these, ordinary provincial capital cities, although being prefecture–level government, have vice–provincial–level chief administrative officers, hence are ranked higher than ordinary prefecture–level cities. Higher administrative levels grant cities priority in resource allocation, development rights, and decision-making, leading to larger populations and economies.

**Fig 7:**
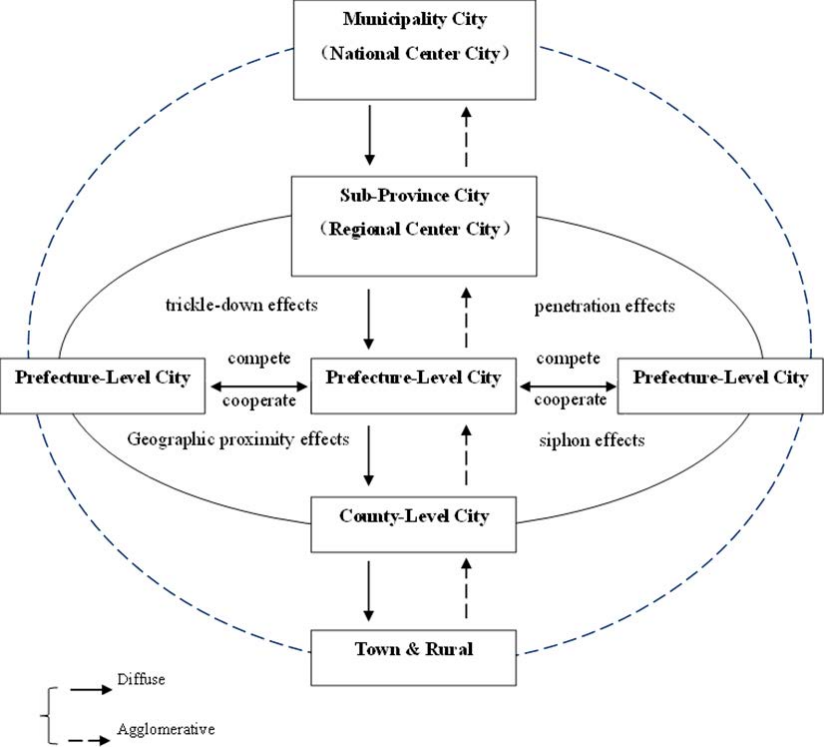
Schematic diagram of a “tiered” structure.

Combining urban agglomeration development patterns with China’s unique conditions shows spatial evolutionary mechanisms and specific administrative systems shape China’s urban agglomeration evolution. Tiered urban agglomerations refer to the presence of an administrative center city within a region, which has a significantly higher administrative level than surrounding cities, where various production factors and resources sequentially flow from higher to lower–level cities under administrative arrangements. Upstream manufacturing and service industries tend to cluster in areas with higher administrative levels, making the administrative center city also the regional core city. Due to restrictions from the household registration and land systems, the dual urban–rural structure and inter–city segmentation hinder the full flow of population and land elements in the hinterlands of urban agglomerations, preventing per capita GDP from achieving equilibrium through aggregation, further exacerbating congestion in central cities and slow development in hinterland cities. On the other hand, spatial evolutionary mechanisms also play a role. According to the agglomeration theory of new economic geography, various enterprises and production factors within a broad region tend to cluster towards the regional center, as agglomeration effects provide substantial positive externalities through sharing, matching, and learning mechanisms [24]. Enterprises can benefit from increasing returns to scale, knowledge, and technology spillovers in clustered areas, where production factors are efficiently allocated at higher–level platforms, thus enhancing factor return rates. The scale of central place agglomeration continues to expand due to the presence of endogenous returns to scale. However, the costs of agglomeration also increase with the degree of clustering, most directly manifested as a series of issues such as high living costs, urban congestion, increased crime rates, and market failure associated with “big city diseases.” When some enterprises, families, and labor within the central city cannot bear the costs brought by clustering, they migrate to peripheral areas, forming new living and production zones on the outskirts of the central city. Under the combined forces of these two mechanisms, the spatial form of China’s urban agglomerations has evolved into a tiered structure with the administrative center as the core city, surrounded and nested by mid and low–level cities closely interconnected (Fig 7). This city network, relying on metropolises and encompassing surrounding medium and small cities, more closely resembles the metropolitan areas of the United States and Japan in spatial form. Therefore, this paper posits that China’s urban agglomerations are still in an intermediate stage transitioning from a monocentric metropolitan circle to a polycentric urban agglomeration, exhibiting a tiered spatial structure.

### Theoretical mechanisms and research hypotheses on the tiered spatial structure of urban agglomerations

The tiered spatial structure of urban agglomerations has three key implications. First, a portion of the population and economic functions of core cities diffuse to surrounding mid–sized cities, enhancing their competitiveness, siphoning capacity, and development potential, thereby transforming them into sub–central cities in the region. Several small metropolitan areas, with core and sub–central cities as growth poles, collectively form a closely connected “competitive–cooperative” large regional layer. Second, the development potential energy differential among member cities within the urban cluster transforms into kinetic energy, driving the growth of peripheral small– and medium–sized cities. Core cities leverage the trickle–down and diffusion effects to activate spatial transmission mechanisms, allowing positive spatial externalities to spill over and stimulate the development of surrounding cities. Third, by relying on the urban cluster, an industrial system with a reasonable gradient is established. Core cities continuously upgrade industries toward capital–intensive and technology–intensive sectors, while relatively lagging industries relocate to peripheral areas. By absorbing mid– and downstream industries, peripheral cities not only mitigate urban challenges faced by core cities but also enhance their own development efficiency, promoting the coordinated development of the entire region through the synergistic “scale borrowing” method between cities. These implications significantly influence the region’s economic resilience.

#### Mechanisms of population and economic polycentricity’s impact on regional economic resilience.

The increasing returns to scale generated by agglomeration create positive externalities that promote economic growth; however, there is an inverted “U–shaped” relationship between the degree of agglomeration and economic growth. Excessive agglomeration leads to an “agglomeration shadow,” where the externalities of economic growth shift from positive to negative [25–27]. A monocentric spatial structure creates agglomeration shadows, and as agglomeration costs rise, it weakens the positive externalities of agglomeration, resulting in “big city diseases” such as urban congestion, environmental pollution, and declining per capita social welfare [28]. A monocentric spatial structure can lead to an agglomeration shadow. As agglomeration costs rise, the positive externalities of agglomeration diminish, leading to “big city diseases” like urban congestion, environmental pollution, and a decline in per capita social welfare. Conversely, a monocentric structure exerts a strong siphoning effect on surrounding areas, causing an excessive concentration of resources and high–quality production factors. Peripheral cities experience “stalled” and “disabled” development. Combined with structural asset disparities and the wealth ratchet effect, this ultimately leads to the “Matthew Effect,” where regional development falls into the “Lucas Paradox,” hindering convergence among cities, undermining the region’s sustainable development, and weakening the urban cluster’s ability to resist external shocks, recover adaptively, and undergo evolutionary transformation.

The polycentric spatial structure of urban agglomerations can enhance regional labor productivity and land use efficiency [29], effectively mitigating the excessive siphoning and high agglomeration costs associated with unipolarity. By leveraging the diffusion and reflow effects of secondary cities, it promotes broader hinterland development [30,31], thereby significantly boosting regional economic resilience [32]. A polycentric spatial structure in urban agglomerations can effectively mitigate excessive siphoning and high agglomeration costs caused by monopolarization, while also promoting broader hinterland development through the diffusion and backflow effects of sub–central cities. China has a large population, and within urban agglomerations, multiple population centers already exist; however, economic centers are relatively singular, leading to a spatial mismatch between population and economic development. Population center cities have substantial demand for consumption, investment, and trade, and the government has more policy tools and investment capacity to stimulate recovery from recessions. During economic shocks, the tertiary sector, basic manufacturing, and government investment can still provide substantial employment opportunities. Economic center cities, possessing a more comprehensive range of industries, more competitive industries, and higher–end services, are better equipped to resist risks, recover, and evolve. They play an “Stabilizing Mechanism” role within the region, driving peripheral areas to undergo industrial upgrading and transformation, creating jobs and investments. Both forms of centrality bolster regional economic resilience. Some scholars compared the effects of two types of polycentric structures on economic growth and found that economic polycentricity exerts a more pronounced positive impact on regional economic growth [33]. However, in China, population center cities often lack a high level of economic development, meaning that population centers are not necessarily economic centers. Therefore, this study must separately examine the impact of economic (functional) polycentricity and population (morphological) polycentricity on regional economic resilience, and further explore the effects of the overlap between these two types of centers.

Based on the foregoing discussion, this paper proposes Hypothesis H1: A regional spatial structure characterized by both population polycentricity and economic polycentricity enhances the economic resilience of urban agglomerations, and the degree of coupling between these two centers further positively impacts regional economic resilience.

#### The impact mechanism of the “core-periphery” development gap on regional economic resilience in urban agglomerations.

**Fig 8:**
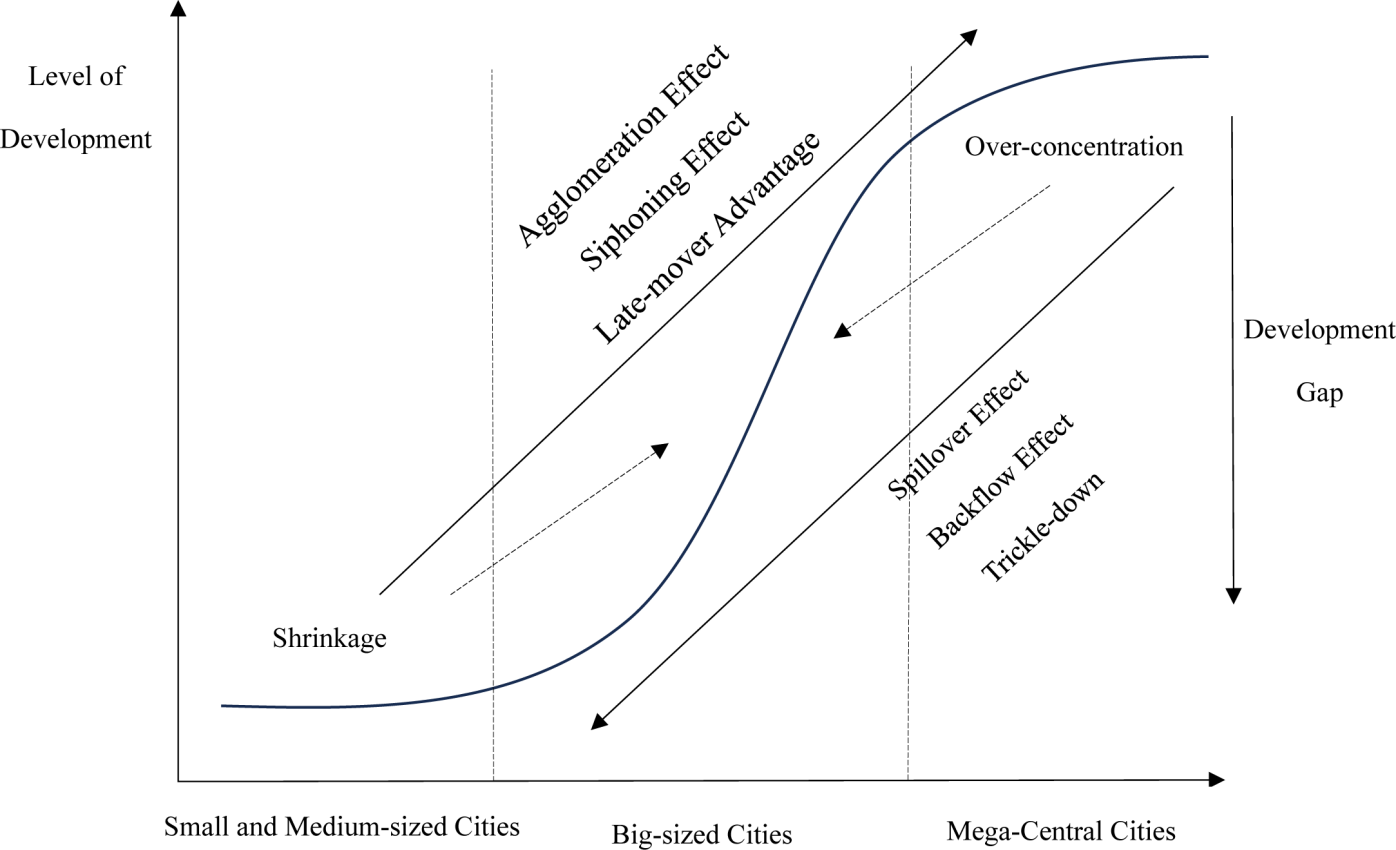
Diagram of concentric urban agglomeration spatial mechanism (before).

**Fig 9:**
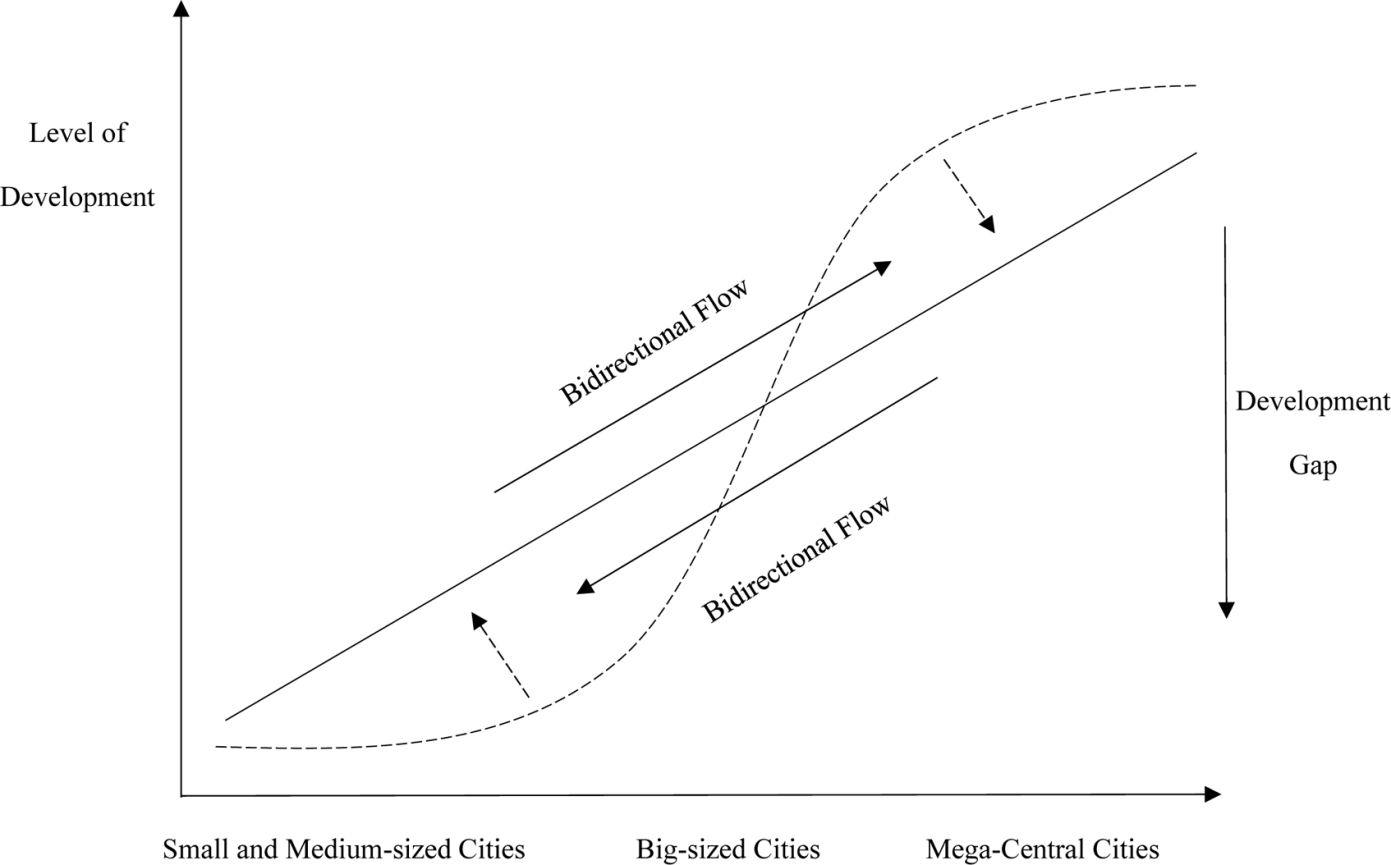
Diagram of concentric urban agglomeration spatial mechanism (after).

China’s regional development gap is evident in three dimensions: coastal vs. inland areas, large cities vs. small cities, and urban vs. rural areas. During economic fluctuations, large-scale macro-regions, due to significant differences in economic structures, development stages, and growth drivers, experience slow and inefficient market adjustments, rendering them unsuitable as optimal targets for policy regulation. This results in an inability to promptly and comprehensively adjust to resist risks. To address regional development imbalances, the Chinese government has implemented coordinated development and new urbanization strategies, leveraging urban agglomerations to promote convergence in development levels between core large cities and peripheral small– and medium–sized cities. Simultaneously, emphasis is placed on cultivating the primary functions of each city and shaping intercity relationships with smooth development gaps, effectively harnessing spatial transmission mechanisms such as agglomeration–diffusion and siphoning–trickle effects (Fig 8 and Fig 9).

The spatial scale of urban agglomerations falls between the macro and micro levels, characterized by a high degree of integration among member cities, forming a relatively close-knit regional network. This interactive network can promote the economic growth of member cities [34]. The spatial scale of urban agglomerations falls between macro and micro levels, with a high degree of integration among member cities, collectively forming a tightly connected regional network system. Throughout the urbanization process, China has implemented a large–city development–oriented regional policy, allowing regions with first-mover advantages to rapidly develop into core growth poles at both national and regional levels through capital accumulation and policy intervention, while surrounding small- and medium-sized cities remain underdeveloped. The development gap within urban agglomerations can enable the high development potential of advanced areas to be converted into growth momentum for less developed areas. The development gap within an urban agglomeration enables the high development potential of advanced areas to be transformed into development momentum for less developed areas, promoting convergence between cities and strengthening the regional economic resilience of the urban agglomeration [35]. The high potential for development in core cities can be transformed into developmental momentum for hinterland cities through the effects of industrial relocation, human capital return, and the diffusion of technology and knowledge, further stimulating endogenous forces through latecomer advantages and comparative advantages in trade. Medium and small cities in the hinterlands of urban agglomerations can “borrow” the high–end service functions and scale economy spillover effects of nearby large cities, obtaining positive spatial externalities while avoiding the costs of agglomeration and optimizing endogenous growth drivers. Under the influence of these two forces, medium and small cities within urban agglomerations can maintain relatively high economic growth rates, creating more jobs, larger investment spaces, and more diversified trade cooperation, all of which play a significant role in enhancing economic resilience.

Building on the preceding discussion, this paper presents Hypothesis H2: The development gap within the “core–periphery” structure of urban agglomerations contributes to enhancing the overall economic resilience of the cluster.

#### The impact mechanism of industrial gradient between core cities and peripheral cities on regional economic resilience.

Industry is the foundation of national economic growth and public well–being. An imbalanced regional industrial structure can undermine regional economic resilience. When facing external economic shocks, regions with concentrated industries tend to experience lower unemployment rates, but excessive concentration can weaken the overall region’s or nation’s ability to resist unemployment [36]. Coordinated development of urban agglomerations within a layered structure not only facilitates the flow of production factors but also promotes a more balanced spatial distribution of industrial structures and value chains. This approach empowers the industrial development of small– and medium–sized cities and enhances the industrial sophistication of core cities while rationalizing the overall regional industrial gradient. Studies have shown that similar industrial structures can result in homogenized competition and risk diffusion, weakening the economic resilience of metropolitan areas. In contrast, diverse industrial systems [37] and refined regional industrial specialization [7] have a significant positive impact on regional economic resilience. Currently, China’s regional industrial structure imbalance faces issues of intercity industrial mismatch, stemming from the singular and weak industrial structure of small– and medium–sized cities, poorly integrated industrial linkages between cities, and the inadequate diffusion and driving effects of core cities’ industrial systems. When there is a mismatch between the industrial structures of core and peripheral cities, the latter are unable to provide the supporting industries, talent, capital, and intermediate products needed by the core cities. They may also be constrained by the path dependence and lock–in of traditional industries, unable to achieve industrial transformation and upgrading in the short term. In this scenario, core cities’ high–end services and upstream enterprises might seek external partnerships, raising resource and factor mobility costs. This results in missed opportunities for urban agglomeration’s later–developing cities, widening the development gap and leading to a cycle of mismatch and missed opportunities.

Leveraging the layered spatial structure of urban agglomerations to promote a rational industrial gradient allows for the economic absorption of industries and production factors relocated from core cities, while also deepening intercity cooperation. Peripheral cities can alleviate the problems of core cities by “scale borrowing,” diverting non–core functions from the core cities. This process not only reduces excesses, enhances efficiency, and empowers the development of large cities, but also drives the prosperity of small– and medium–sized cities through spatial diffusion, promoting the coupling of industrial gradients with spatial structures and achieving the rationalization and upgrading of industrial structures, strengthens the region’s ability to withstand external shocks and recover afterward [38]. Enabled by modern transportation, information, and logistics networks, the low–end industries of small– and medium–sized cities can integrate into the regional market, promoting the fusion of their primary, secondary, and tertiary industries. This integration enables path–breaking advancements and the transformation of growth drivers, thereby enhancing development levels and increasing the overall economic resilience of the region.

**Fig 10:**
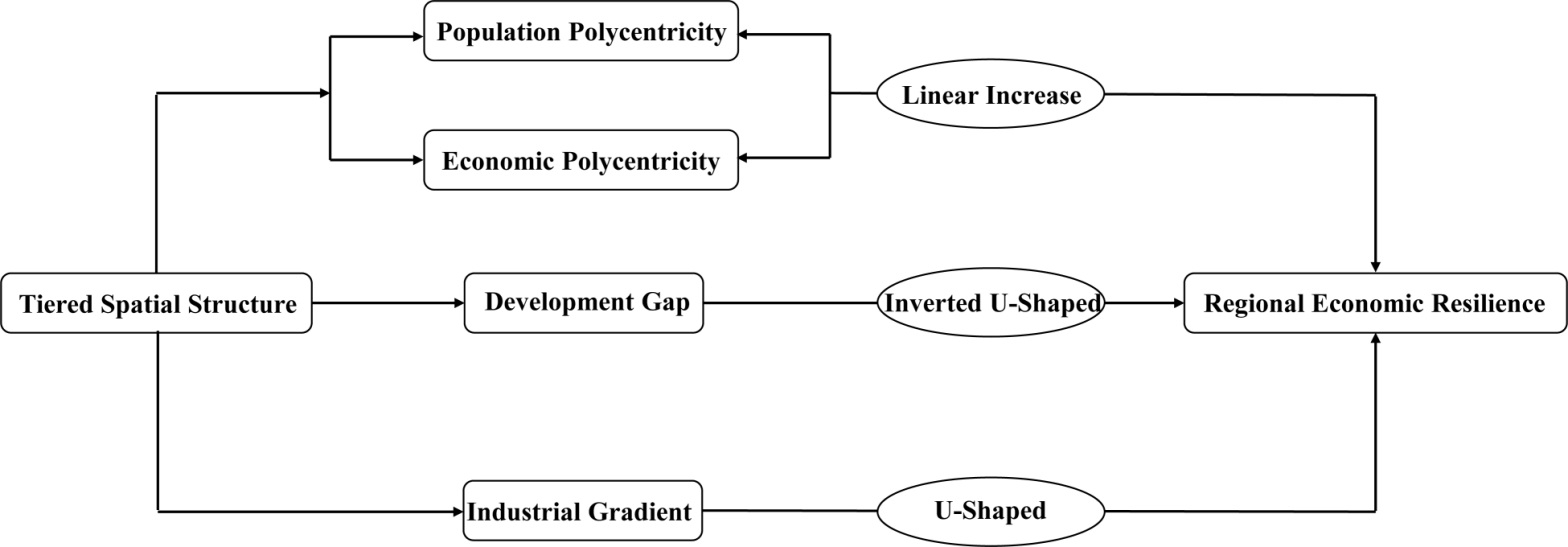
Theoretical mechanism diagram.

Based on the above, this paper proposes Hypothesis H3: The more rational the industrial gradient between core and peripheral cities, the stronger the regional economic resilience (Fig 10).

## Research design

### Econometric model

Based on the mechanisms of tiered spatial structures mentioned above and the research hypotheses established, this study explores the impact of urban agglomeration tiered structures on regional economic resilience from the perspectives of urban agglomeration polycentricity, the “core–hinterland” economic development gap, and industrial gradient. The article introduces the first–order lag of the dependent variable as one of the explanatory variables, based on dynamic panel data, with the econometric model as follows:


$$RES_{it}=\beta_{0}+\beta_{1}POP_{k}+\beta_{2}ENT_{k}+\beta_{3}POP_{K}*ENT_{k}+\beta_{4}RES_{it-1}+\overset{n}{\underset{i=1}{\sum }}\alpha_{i}C_{i}+\mu_{it}+\eta_{it}+\varepsilon_{it}$$ (1)

$$RES_{it}=\beta_{0}+\beta_{1}GAP_{k}+\beta_{2}RES_{it-1}+\overset{n}{\underset{i=1}{\sum }}\alpha_{i}C_{i}+\mu_{it}+\eta_{it}+\varepsilon_{it}$$ (2)

$$RES_{it}=\beta_{0}+\beta_{1}GRAD_{k}+\beta_{2}RES_{it-1}+\overset{n}{\underset{i=1}{\sum }}\alpha_{i}C_{i}+\mu_{it}+\eta_{it}+\varepsilon_{it}$$ (3)

Herein, i and t respectively denote the individual and time. $RES_{it}$ represents the economic resilience of urban agglomerations, $RES_{it-1}$ denotes the first lag of the explanatory variable, $POP_{k}$represents the polycentricity of population in city cluster k, $ENT_{k}$denotes the economic polycentricity, $POP_{K}*ENT_{k}$is the interaction term, $C_{i}$represents control variables, $\mu_{it}$denotes individual fixed effects, $\eta_{it}$represents time fixed effects, and $\varepsilon_{it}$denotes the error term.

Given the potential bidirectional causality between regional economic resilience and polycentricity, the “core–periphery” gap, and industrial gradients, as well as other endogeneity issues such as omitted variables, the System GMM approach is employed in the empirical analysis to address endogeneity. System Generalized Method of Moments (GMM) uses predetermined and lagged values of endogenous variables as instruments, resolving the issue of endogeneity in dynamic panel data.

### Variables

#### Dependent variables.

Regional Economic Resilience. Common measures of regional economic resilience in existing research include composite indices based on economic growth rates, employment or unemployment data, sensitivity, and ratio methods. For instance, Martin et al. (2016) use the sensitivity of economic growth rates to assess urban resilience [7]; Mai et al. (2021) evaluate a city’s relative resilience through the difference and ratio of local to national GDP growth rates [10]; Chinese scholar Xiong (2023) employs a composite index method to measure urban resilience across ecological, economic, social, and engineering dimensions [39]. This paper adopts Martin (2016)’s method for measuring urban economic resilience, assessing overall economic resilience by the difference between actual and expected changes in the gross domestic product of various urban agglomerations:


$$RES_{it}=\frac{\Delta E_{t-1}^{t}-\Delta E_{i}^{t}{}^{\text{e}xp}}{\left|\Delta E_{i}^{t}{}^{\text{e}xp}\right|}$$ (4)

$$\Delta E_{i}^{t}{}^{\text{e}xp}=g_{N}^{t}E_{i}^{t}$$ (5)

In Eq (4), $\Delta E_{i}^{t}$ denotes the difference in the gross regional product of urban agglomeration i between year t and t–1, while $\Delta E_{i}^{t}{}^{\text{e}xp}$ represents the expected gross regional product of urban agglomeration i in year t; in Eq (5), $g_{N}^{t}$ denotes the national GDP growth rate in year t, and $E_{i}^{t}$ represents the gross regional product of urban agglomeration i in year t. A higher value of regional economic resilience, $RES_{it}$, indicates that the actual change in the gross product of the area exceeds the expected change calculated based on the national GDP growth rate, signifying stronger resilience, and vice versa.

#### Main explanatory variables.

(1) Polycentricity. Current academic measures of polycentricity primarily focus on morphological polycentricity, functional polycentricity, and comprehensive measures. Notably influential methods include the composite index measures used by the POLYNET project and ESPON project, which equate urban polycentricity with a networked urban system. Researchers such as Keyun Zhang (2022) measure the morphological polycentricity of cities using nighttime light data [22]. Meijers et al. (2018) calculate urban polycentricity using the HHI index [21]. Meijers and Burger (2010) use the Pareto index of city size distribution to measure polycentricity within larger regions [40]. Dongyang Zhang et al. (2023) and Peng (2023) measure morphological polycentricity using population data [18,19]. while Li et al. (2017) assess functional polycentricity using the city functional linkage ranking index [41]. This paper measures the polycentricity of population ($POP_{k}$) and economy ($ENT_{k}$) of cities based on the year–end permanent population and the number of large–scale industrial enterprises, using the Four–City Index method. The specific formula is as follows:


$$POP_{k}=\frac{P_{1}}{\sum_{\dot{l}=2}^{4}P_{i}}$$ (6)

$$ENT_{k}=\frac{E_{1}}{\sum_{\dot{l}=2}^{4}E_{i}}$$ (7)

In Eq (6), P_1_ denotes the population size of the largest city, and P_i_ represents the population size of the ith city, where i ranges from 2 to 4; in Eq (7), E_1_ denotes the industrial enterprise size of the largest city, and E_i_ represents the industrial enterprise size of the ith city, where i ranges from 2 to 4. The values of POP and ENT range from 0 to 1, with higher values indicating a stronger monocentricity, whereas lower values suggest stronger polycentricity. Given the late start of urban agglomeration development in China and the yet–to–be–coordinated population and economic scales among member cities, characterized by a dual–center feature, an interaction term between the two is introduced to measure the coupling degree of population and economic polycentricity.

(2) Core–Periphery Development Gap. The development gap is widely applied in regional economic studies, with common quantification methods including the index method based on comprehensive measures [42], the Gini coefficient method based on GDP or per capita GDP [43], and the ratio/difference method [44]. This paper adopts the difference method based on per capita GDP to measure the economic development gap between core cities and hinterland cities, calculated by subtracting the average GDP of other member cities from the per capita GDP of the leading city within the urban agglomeration. The specific formula is as follows:


$$GAP_{k}=G_{k1}-\bar{G_{k}}$$ (8)

Herein, G_k1_ denotes the per capita GDP of the leading city in city cluster k, while $\bar{G_{k}}$represents the average per capita GDP of the hinterland cities in city cluster k. A larger value of the “Core–Periphery” development gap, GAP, for city cluster k indicates a greater economic development disparity between the core city and hinterland cities, and vice versa.

(3) Core–Hinterland Industrial Gradient. In numerous academic studies on resilience from an industrial perspective, it is common to measure urban industrial structure and high–end industries using the ratio of secondary to tertiary sectors, or to assess urban industrial diversity and agglomeration with the HHI index, VAR index, or location entropy [9,45,46]. Scholars also use the industrial gradient coefficient to measure the level of industrial gradient within a region. This paper focuses on urban agglomerations and uses the ratio of the employment percentage in the secondary sector of hinterland cities to the employment percentage in the tertiary sector of the core leading city to measure the internal industrial gradient within urban agglomerations. The specific formula is as follows:


$$GRAD_{k}=\frac{INT_{k2}∕INT_{k}}{CEN_{k3}∕CEN_{k}}$$ (9)

Herein, INT_k2_ denotes the average number of employees in the secondary industry of hinterland cities in city cluster k, INT_k_ represents the total number of employees across all industries in the hinterland cities of city cluster k, CEN_k3_ denotes the number of employees in the tertiary industry of the core city in city cluster k, and CEN_k_ represents the total number of employees across all industries in the core city of city cluster k. A larger “Core–Hinterland” industrial gradient, GRAD, for city cluster k indicates a more gradual industrial gradient, suggesting a more rational industrial structure within the region, and stronger industrial absorption capacity, and vice versa.

#### Control variables.

The control variables selected for the study include: scale of foreign capital utilization, telecommunications services scale, level of economic development, government intervention, fixed asset investment, consumer market scale, and public services.

Foreign Capital Utilization Scale (FDI): Rational use of foreign capital is an important indicator of the Chinese government’s administrative performance and a key factor in promoting China’s rapid economic development over the past decades. The study uses the actual amount of foreign capital utilized. Telecommunications Services Scale (Tel): The digital economy and information services, as emerging economic sectors, are vital pathways for economic transformation and upgrading and new points of economic growth. The study measures this using the total volume of telecommunications services. Level of Economic Development (Ave GDP): Regions with higher levels of economic development generally have more comprehensive and higher–end industries, better infrastructure, etc., which indirectly affect economic resilience. Considering the potential “big but not strong” aspect of economic development, the study uses per capita GDP to reflect the level of economic development. Government Intervention (Gov): Government fiscal expenditure aims to ensure people’s livelihoods, regional coordinated development, green and low–carbon development, technological innovation, etc. The study represents this with general budget expenditures of local finances. Fixed Asset Investment (Inv): Investment is one of the main factors directly affecting economic growth. The study uses the total fixed asset investment of the whole society to represent this. Consumer Market Scale (Con): Consumption is a major driver of economic growth. With the development of the digital economy and new business formats, the contribution rate of consumption to economic growth has gradually increased. The study uses the total retail sales of consumer goods to represent this. Public Services (Pub): High–quality public services can enhance regional human capital and are an important indicator for attracting population settlement. The study uses the number of doctors and teachers per ten thousand people as indicators.

### Sample and data

**Fig 11:**
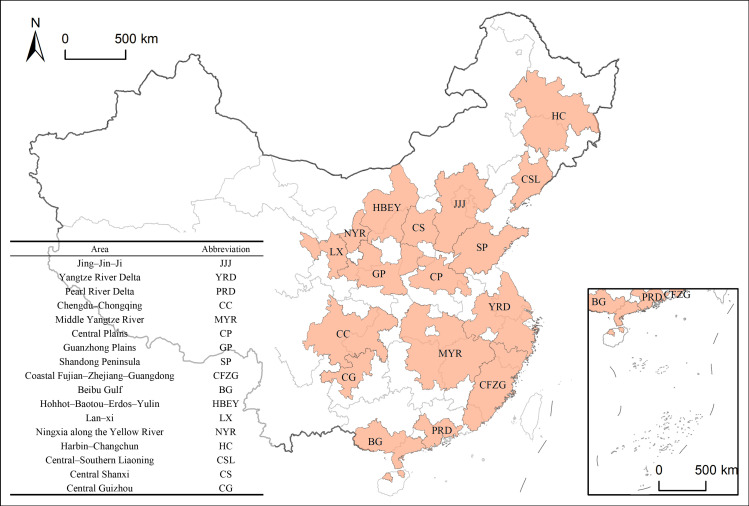
Research area. Note: The vector data for China's administrative boundaries were obtained from the Standard Map Service website of the Ministry of Natural Resources of China (http://211.159.153.75/index.html).

China’s 14th Five–Year Development Plan identifies 19 national urban agglomerations, with 17 chosen for this study: Jing–Jin–Ji, Yangtze River Delta, Pearl River Delta, Chengdu–Chongqing, Middle Yangtze River, Central Plains, Guanzhong Plains, Shandong Peninsula, Coastal Fujian–Zhejiang–Guangdong, Beibu Gulf, Hohhot–Baotou–Erdos–Yulin, Lan–Xi, Ningxia along the Yellow River, Harbin–Changchun, Central–Southern Liaoning, Central Shanxi, and Central Guizhou (Fig 11). The Northern Slope of Tianshan and Central Yunnan were excluded due to their ethnic region locations and data limitations.

**Fig 12:**
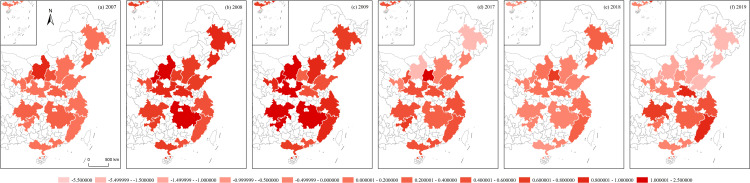
RES of Chinese urban agglomerations.

**Fig 13:**
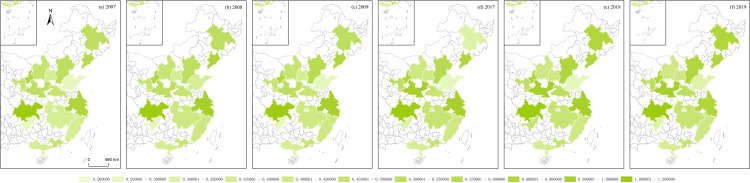
POP of Chinese urban agglomerations.

**Fig 14:**
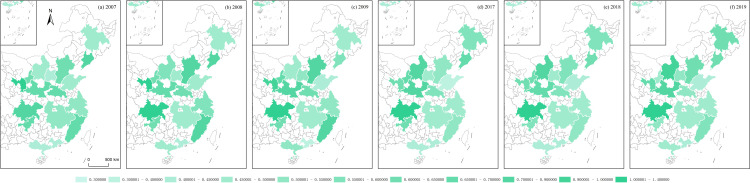
ENT of Chinese urban agglomerations.

**Fig 15:**
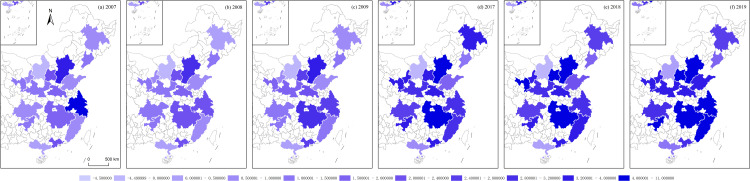
GAP of Chinese urban agglomerations.

**Fig 16:**
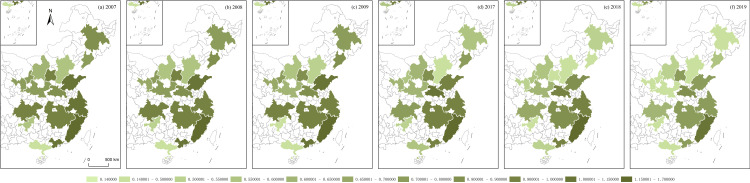
GRAD of Chinese urban agglomerations. Note: The vector data for China's administrative boundaries were obtained from the Standard Map Service website of the Ministry of Natural Resources of China (http://211.159.153.75/index.html).

The geographic spatial data used in this study were sourced from public platforms and do not involve any intellectual property disputes. The vector data for China’s administrative boundaries in Figs 11–16 were obtained from the Standard Map Service website of the Ministry of Natural Resources of China (http://211.159.153.75/index.html). The street maps in Fig 11 were sourced from the National Geoinformation Public Service Platform of China (https://vgimap.tianditu.gov.cn/).

The study period is set from 2005 to 2019, due to the “China City Yearbook” lacking industry employment data for 2020 and 2021 and inconsistencies in some variables’ measurements before 2005. The final study sample is determined as the 17 national–level urban agglomerations from 2005 to 2019.Data for various variables are derived from the “China City Statistical Yearbook,” “China Regional Economic Statistical Yearbook,” statistical bulletins of prefecture–level cities, and statistical yearbooks of provinces and cities, with China’s annual inflation rate data obtained from the World Bank. Descriptive statistics of the variables are shown in Table 1.

**Table 1:** Descriptive statistics of variables (2005–2019).

Variable	Obs	Mean	Std	Min	Max
RES	255	0.268	0.830	–5.485	2.442
POP	255	0.536	0.203	0.228	1.160
ENT	255	0.601	0.187	0.318	1.372
POP*ENT	255	0.341	0.234	0.097	1.359
GAP	255	2.030	1.925	–4.426	10.677
GRAD	255	0.808	0.263	0.144	1.749
LnFDI	255	3.734	1.695	–0.626	6.557
LnTel	255	5.863	1.080	2.639	7.751
Avegdp	255	4.448	2.595	0.607	12.257
Gov	255	0.159	0.053	0.068	0.286
Inv	255	0.685	0.231	0.247	1.282
Con	255	1.710	1.001	0.211	4.867
LnPub	255	4.747	0.126	4.459	5.052

### Analyzing trends in dependent and independent variables within Chinese urban agglomerations

Using the algorithms of the aforementioned variables, this section illustrates the regional economic resilience, population polycentricity, economic polycentricity, “core–periphery” city development gap, and industrial gradient for 17 urban agglomerations during the periods 2007–2009 and 2017–2019. It analyzes the performance of regional economic resilience in Chinese urban agglomerations under the impacts of the 2008 global financial crisis and the 2018 China–U.S. trade conflict, along with the changing trends of each variable during these periods.

During the global financial crisis, the regional economic resilience of Chinese urban agglomerations in 2008 generally strengthened, exhibiting strong resistance. In 2009, the first year of recovery after the shock, regional economic resilience generally weakened in northern Chinese urban agglomerations, except in a few, such as Beijing–Tianjin–Hebei, while southern urban agglomerations generally strengthened. However, from 2009 to 2017, during this relatively long period, the economic growth rate of Chinese urban agglomerations generally slowed, and the corresponding regional economic resilience also weakened. During the China–U.S. trade conflict, the regional economic resilience of Chinese urban agglomerations in 2018 generally weakened. In 2019, the first year of recovery after the shock, regional economic resilience in northern Chinese urban agglomerations further weakened, except in the Central Plains urban cluster, which showed weaker recovery capacity, while southern urban agglomerations generally experienced a rebound in economic growth, demonstrating stronger recovery capacity. Observing the trends in population polycentricity, economic polycentricity, “core–periphery” city development gap, and industrial gradient in Chinese urban agglomerations from 2007 to 2019 reveals that in most northern urban agglomerations, population and economic monopolar agglomeration intensified, while southern urban agglomerations exhibited a polycentric development pattern in both population and economic agglomeration. The development gap between cities in eastern urban agglomerations gradually converged, while the gap further widened in the central, western, and northeastern urban agglomerations. The overall trend in industrial structure showed greater rationalization, with eastern urban agglomerations outperforming those in the central and western regions (Figs 12–16).

## Empirical test

### Main regression

The fixed effects model was selected, based on the Hausman test, to improve estimate precision and address heteroscedasticity across cities and periods. Models M1–M3 present the main regression results, highlighting the significance of the dependent variable’s first lag. The Arellano-Bond test accepted AR(2) at the 10% confidence level, indicating the absence of second-order serial correlation. The Hansen test results were all above 0.1, confirming the validity of the over-identification of GMM instruments. specific estimates are in Table 2. M1 details regression results on how population and economic polycentricity, and their interaction, affect regional economic resilience. Significant negative correlations were found between both population polycentricity and economic polycentricity with economic resilience at the 5% and 10% levels, suggesting that greater concentration within a city cluster weakens economic resilience. The interaction term positively correlates with resilience at the 1% level, showing that tighter integration of population and economic centers boosts economic resilience, supporting H1. M2 reports the regression results on the impact of the development gap between core cities and hinterland cities on regional economic resilience. M3 finds a significant positive correlation at the 10% level between the industrial gradient and economic resilience, showing that a higher secondary–to–tertiary industry ratio in hinterland versus core cities boosts resilience, supporting H3.

**Table 2:** Main regression and robustness results.

variable	M1	M2	M3	M4	M5	M6
*L.Ln* $RES_{it}$	–0.347***	–0.250*	–0.381***	–0.484***	–0.328*	–0.312**
	(0.134)	(0.144)	(0.120)	(0.158)	(0.170)	(0.140)
POP	–2.085**			–2.363*		
	(0.906)			(1.374)		
ENT	–2.717*			–2.882*		
	(1.556)			(1.607)		
POP*ENT	3.561***			3.708**		
	(1.326)			(1.555)		
*GAP*		0.152*			0.262*	
			(0.078)			(0.153)
*GRAD*			0.560*			1.018*
			(0.328)			(0.586)
control variable	control	control	control	control	control	control
ID/Time	control	control	control	control	control	control
AR(2)	0.113	0.108	0.104	0.220	0.244	0.308
Hansen P	0.262	0.153	0.190	0.158	0.939	0.338
_cons	335.5	431.1**	–32.13	176.1	293.0*	390.4
	(218.829)	(199.204)	(203.724)	(224.025)	(168.864)	(248.389)
*N*	238	238	238	170	170	170

Standard errors in parentheses.

*p <  0.1,

**p <  0.05,

***p <  0.01.

### Robustness tests

Given a small dynamic panel dataset with N (17) >  T (15), this study utilizes a one–step system GMM method for reliable estimation and employs robust standard errors to reduce omitted variable bias. The study controls for individual (urban agglomerations) and time (year) fixed effects for data stationarity and heteroscedasticity, applying logarithmic transformations and decimal adjustments to some indicators.

Table 2’s models M4, M5, and M6 use data from 2009 to 2019, excluding years before 2008. This approach considers the significant impact of the 2008 global financial crisis, focusing on regression outcomes in a more stable environment post–shock. The core explanatory variables’ signs and significance levels stay consistent, aligning with the main regression findings and confirming result robustness. Moreover, regression model (1) analyzes regional polycentric structures’ effects on economic resilience, including both population and economic polycentricity variables. The consistent sign and significance of these variables further underscore the regression outcomes’ robustness.

### Further research

To explore the impact of the layered spatial structure of urban agglomerations on regional economic resilience and determine the optimal value range of the explanatory variables, this paper employs a threshold regression model to further examine the threshold effect of the aforementioned main research conclusions. After conducting a threshold effect test on the independent variables, it was found that population polycentricity (POP) and economic polycentricity (ENT) do not exhibit threshold effects beyond the first order, while the development gap (GAP) between core and peripheral cities and the industrial gradient (GRAD) exhibit double threshold effects (Table 3).

**Table 3:** Threshold effect significance test.

Dependent Variable	Threshold Variable	Type Test	F-Value	P-Value
		Single Threshold	18.14**	0.03
RES	GAP	Double Threshold	14.80*	0.063
		Triple Threshold	6.66	0.39
		Single Threshold	13.52**	0.0133
RES	GRAD	Double Threshold	10.90*	0.0153
		Triple Threshold	6.37	0.520

As shown in Table 4, the threshold values for the development gap between core and peripheral cities are -2.77 and 3.96, respectively. When the development gap is in the range of GAP < -2.77 or -2.77 < GAP <  3.96, the regression coefficient initially increases positively and then negatively as the development gap widens, with significance at the 1% and 5% levels, respectively. It is evident that the impact of the development gap between core and peripheral cities on regional economic resilience exhibits an inverted “U” shape, where the influence increases first and then decreases. The threshold values for the industrial gradient between core and peripheral cities are 0.496 and 0.4407, respectively. When the industrial gradient is in the range of GRAD >  0.496 or 0.496 < GRAD <  0.4407, the regression coefficient shifts from negative to positive as the industrial gradient becomes more rational, with significance at the 5% level. It is evident that the impact of the industrial gradient between core and peripheral cities on regional economic resilience follows a “U” shape, where the influence decreases first and then increases.

**Table 4:** Threshold regression results.

Variables	M7	Variables	M8
GAP(GAP< < -2.77)	0.31***	GRAD(GRAD > 0.496)	11.542**
	(0.113)		(5.390)
GAP(-2.77< < GAP< < 3.96)	-0.185**	GRAD(0.496< < GRAD< < 0.4407)	-2.225**
	(0.082)		(0.998)
GAP(GAP> > 3.96)	-0.159	GRAD(GRAD< < 0.4407)	-1.091*
	(0.186)		(0.584)
_cons	1.096	_cons	0.066
	(4.046)		(3.77)
N	238	N	238
R^2^	0.3528	R^2^	0.3556

Standard errors in parentheses.

*p <  0.1,

**p <  0.05,

***p <  0.01.

### Heterogeneity test

Urban agglomerations and their member cities in China exhibit significant heterogeneity, with diverse reasons contributing to the formation of multi–centered urban agglomerations. Two primary pathways characterize the multi–centric development model of Chinese urban agglomerations. The first pathway sees core cities driving hinterland development, with strong cultural and economic ties fostering regional identity and integrated urban agglomerations. The second pathway arises from significant geographical or administrative separations, enabling cities to develop more independently. These cities rely on modern transportation and communication networks to establish markets for factors, goods, and services, thereby constituting regional urban agglomerations. Moreover, the urbanization process in China spatially evolves with two characteristics: a gradient from the coast to the inland and from regional core cities to hinterland cities. Early this century, China’s role as the “world’s factory” and its export–driven growth model created a spatial pattern of coastal development surpassing inland areas. Thus, it is necessary to discuss the impact of various explanatory variables on regional economic resilience under conditions of coastal location. Furthermore, the national government identified nine national central cities in urban system planning in 2010 and 2016, which enjoy a high degree of resource allocation rights within their regions under the administrative hierarchy system, serving as centers and hubs for finance, management, science and innovation, and transportation at both regional and national levels, and acting as gateways for international exchange and interaction. Therefore, it is essential to discuss the impact of various explanatory variables on regional economic resilience in regions that already have national central cities.

#### Considering the heterogeneity of coastal urban agglomerations.

Table 5 shows regression outcomes for coastal urban agglomerations in models M9–M11 and for inland agglomerations in M12–M14, with significant lagged dependent variables in all models. For coastal areas, population and economic polycentricity positively correlate with economic resilience at 10% and 1% levels, respectively, while their interaction negatively correlates at the 5% level, opposing the main findings. In inland regions, population polycentricity negatively affects resilience at the 5% level, with a positive correlation for the interaction term at the same level, aligning with main findings. Economic polycentricity’s negative impact is not significant. The development gap and industrial gradient between core and hinterland cities show a significant positive correlation in both region types, supporting H2 and H3 and aligning with main results.

**Table 5:** Heterogeneity regression results by coastal status.

variable*	M9	M10	M11	M12	M13	M14
*L.Ln* $RES_{it}$	–0.312***	–0.272***	–0.299**	–0.394*	–0.305*	–0.454**
	(0.065)	(0.049)	(0.143)	(0.231)	(0.171)	(0.226)
POP	5.841*			–3.123**		
	(3.500)			(1.584)		
ENT	7.056***			–1.582		
	(2.595)			(1.280)		
POP*ENT	–12.31**			3.758**		
	(5.034)			(1.570)		
*GAP*		0.129**			0.368*	
		(0.057)			(0.196)	
*GRAD*			2.044**			2.255*
			(0.931)			(1.223)
control variable	control	control	control	control	control	control
ID/Time	control	control	control	control	control	control
AR(2)	0.262	0.297	0.233	0.137	0.875	0.206
Hansen P	1	1	1	1	1	1
_cons	–291.7	–513.9	–1748.9	–308.2	817.4*	1324.5**
	(364.150)	(923.483)	(1412.200)	(288.623)	(449.250)	(601.745)
*N*	98	98	98	140	140	140

Standard errors in parentheses.

*p <  0.1,

**p <  0.05,

***p <  0.01.

Polycentricity and the coupling of population and business centers in coastal urban agglomerations negatively affect the region’s economic resilience. This could stem from the export–oriented growth model favoring China’s eastern coastal regions, resulting in a higher permanent population and more industrial enterprises, and a comprehensive urban and industrial labor division. Large cities within coastal urban agglomerations rapidly grow into national growth poles, with their economic size, industrial scale, and infrastructure and public service scale exceeding their registered population size, possessing a stronger capacity for agglomeration and greater scale benefits. Furthermore, a more specialized division of urban functions leads to the separation of population and economic centers, with labor–intensive industries limited by the high living and production costs in core cities, diffusing back to peripheral hinterland cities, while capital and technology–intensive industries further cluster in major core cities. Therefore, coastal cities at advanced stages of development need to further strengthen population and economic agglomeration, refine urban division of labor, promote regional collaborative development, play the role of growth poles, and enhance regional economic resilience. In contrast, inland urban agglomerations show that population polycentricity and dual–center coupling align with main findings. Economic polycentricity’s insignificance in inland agglomerations may stem from their slower development, reliance on administrative planning, fewer industrial enterprises than coastal areas, and even provincial distribution, minimally impacting their economic resilience. Overall, the polycentric spatial development pattern boosts the region’s resilience.

#### Considering the heterogeneity of urban agglomerations containing national central cities.

Table 6 presents regression outcomes for urban agglomerations with (M15–M17) and without national central cities (M18–M20), noting the first lag of the dependent variable is significant across all models. For urban agglomerations with national central cities, population and economic multicentricity show negative correlations at 10% and 1% levels, respectively, with a positive correlation for the interaction term at 1%. The industrial gradient between core cities and hinterlands positively correlates at 5%, supporting H1 and H3, aligning with main findings. A larger development gap between core cities and hinterlands negatively impacts economic resilience at 5%, suggesting increased disparity lowers resilience. In agglomerations without national central cities, population multicentricity positively correlates at 10%, with no significant findings for economic multicentricity or the interaction term. Both the development gap and industrial gradient between core cities and hinterlands show a significant positive correlation, affirming H2 and mirroring main results.

**Table 6:** Heterogeneous regression results based on the inclusion of national central cities.

variable	M15	M16	M17	M18	M19	M20
*L.Ln* $RES_{it}$	–0.847***	–0.168*	0.150***	–0.305**	–0.404**	–0.511***
	(0.074)	(0.089)	(0.049)	(0.143)	(0.185)	(0.155)
POP	–1.996*			5.402*		
	(1.135)			(3.179)		
ENT	–3.441***			1.916		
	(1.274)			(3.029)		
POP*ENT	4.241***			–7.000		
	(1.091)			(4.977)		
*GAP*		–0.201**			0.422**	
		(0.086)			(0.207)	
*GRAD*			1.789**			1.168*
			(0.757)			(0.638)
control variable	control	control	control	control	control	control
ID/Time	control	control	control	control	control	control
AR(2)	0.127	0.111	0.129	0.134	0.157	0.275
Hansen P	1	1	1	1	1	1
_cons	56.30	–522.8**	672.4	571.3*	1275.6**	–344.5
	(319.541)	(258.281)	(555.420)	(303.576)	(555.542)	(382.826)
*N*	98	98	98	140	140	140

Standard errors in parentheses.

*p <  0.1,

**p <  0.05,

***p <  0.01.

The reduced economic resilience in urban agglomerations with national central cities, due to a widened development gap, can be linked to the cities’ higher administrative levels. In China’s resource distribution system, these central cities have a stronger ability to siphon resources from other cities, causing excessive diversion from hinterlands. Additionally, they often create agglomeration shadows and face urban malaises. If the economic development of surrounding cities is drastically inferior to that of the core cities, hinterland cities may become incapable of accommodating overflow. When part of the population and businesses from core cities choose to relocate due to the inability to bear high costs, it leads to cross–regional outflows of enterprises and populations, causing internal economic efficiency losses and reducing economic resilience. The positive impact of population multicentricity in urban agglomerations without national central cities is due to the lower development and weaker agglomeration of their central cities. These cities have weaker radiating and driving capacities, making regional elements and resources susceptible to being siphoned off by adjacent developed areas, leading to economic development efficiency losses and reduced economic resilience. The insignificant impact of economic multicentricity and dual–core coupling may be due to the cross–regional siphoning of production factors and industrial enterprises within general urban agglomerations. The number and quality of enterprises are inferior to those in developed urban agglomerations with central cities. Factors such as complex geographical environments and suboptimal business conditions lead to the migration of surplus populations, diminishing the significance of economic multicentricity and dual–core coupling effects.

## Conclusion and discussion

China’s urbanization is currently advancing rapidly. Influenced by information and transportation technologies, the urban system is transforming from a relatively independent “point” structure to a “point-axis” and “layered” cluster form. Urban agglomerations have emerged as crucial spatial units for national economic development. The development of urban agglomerations in China is evolving from metropolitan areas to urban clusters. China’s administrative hierarchy influences their spatial structure, exhibiting a “layered” characteristic that profoundly affects spatial connections between cities and the regional economic resilience of urban clusters. This paper analyzes the impact of three factors—the multi-centered spatial structure, the development gap between “core-periphery” cities, and the industrial gradient—on regional economic resilience, grounded in the agglomeration theory of new economic geography and the regional gradient evolution theory of evolutionary economic geography, while considering China’s administrative hierarchy and the spatial heterogeneity of urban clusters. The study finds that the multi-centered distribution of population and economy within urban agglomerations enhances regional economic resilience. The development gap between “core-periphery” cities exhibits an inverted “U” relationship with regional economic resilience, indicating that as the gap increases, regional economic resilience first strengthens and then weakens. The rationality of the industrial gradient between “core-periphery” cities demonstrates a “U” relationship with regional economic resilience, indicating that as the proportion of secondary industry in peripheral cities within the urban agglomeration increases, regional economic resilience first weakens and then strengthens.

To mitigate the impact of endogeneity on the experimental results, this study employs a one–step system Generalized Method of Moments (GMM) regression model. Empirical analyses assessed tiered spatial structures’ effects on economic resilience, considering variables like coastal location and the presence of national central cities. Findings show that urban agglomerations with multicentric populations and economies grow faster than the national average during fluctuations, highlighting the significant positive impact of multicentric structures on economic resilience. This aligns with the conclusions of numerous studies on urban multicentric structures [46,47]. Additionally, the study reveals a significant positive effect of the coupling between population and economic bicentricity on economic resilience. The GDP per capita disparity between core cities and hinterland cities within urban agglomerations also enhances regional economic resilience, suggesting that there should be a reasonable development gradient between cities of different levels. Spatial balance is crucial for resilience; eliminating disparities without discrimination can lower regional economic resilience. This finding is similar to those of studies related to regional coordinated development [48]. However, disparities should not be extreme. A ratio nearing 1 between secondary sector employment in hinterlands and tertiary in core cities correlates with higher economic resilience; otherwise, resilience declines. This suggests that a balanced industrial gradient enhances economic resilience. However, a large development gap can disconnect core cities from their hinterlands. This disconnection, through industrial misalignment and supply chain disruptions, can cause an imbalance in the industrial gradient, leading to the spillover of various regional elements. This diminishes local development, reduces the region’s capacity to withstand external economic shocks and undergo evolutionary transformations, aligning with findings from literature exploring urban resilience from an industrial perspective [28,29]. In studies considering the heterogeneity of regions being coastal or not, it was found that coastal urban agglomerations, due to their higher levels of development, industrial specialization, and regional synergy, are well–suited to strengthening the agglomeration of core cities. Further agglomeration boosts these regions’ competitiveness and resilience. Studies on the heterogeneity of including national central cities within a region reveal that urban agglomerations without national central cities, located in the less developed central and western regions with smaller populations and economic sizes, are suitable for strengthening internal agglomeration to foster core cities. In contrast, urban agglomerations with national central cities need to gradually narrow the development gap between regions to avoid efficiency losses detrimental to regional economic resilience caused by excessive resource siphoning, development disconnects between cities, and agglomeration shadows.

This study, uniquely focusing on urban agglomerations as extensive regional entities, provides fresh empirical insights on economic resilience, setting it apart from research centered on cities or provinces. Additionally, examining developmental gradients between cities reflects the diverse stages of regional development and urbanization in China more accurately. However, the study also faces limitations and regrets, particularly in data acquisition. Since China does not provide direct panel data for urban agglomerations, data had to be aggregated from individual city data. The limited scope of China’s official city–level data and inconsistencies in some metrics presented challenges in setting variables. For instance, data on industry segmentation at the city level was limited to employment figures, presenting certain constraints.

Based on the above conclusions and the distinct characteristics of China’s urban agglomerations, this paper argues that a single regional development strategy cannot be universally applied. Therefore, China’s urban agglomerations must address the following issues in future development: First for inland regions and those with national central cities, developing multicentric urban agglomerations can effectively enhance regional economic resilience against various internal and external shocks. It should also consider relaxing restrictions on the flow of certain elements, such as household registration policies and local market protection policies. This facilitates the gradual coupling of regional population and economic centers, allowing for balanced development in terms of per capita output through inter–regional labor mobility, thereby correcting spatial mismatches in resources. For coastal areas, refining the division of labor between cities, enhancing overall regional synergy, and promoting the further agglomeration of high–end services and high–skill, high–return elements in core cities can transform coastal urban agglomerations into highly integrated and internationally competitive entities. For inland areas without central cities, leveraging the externalities spillover from developed areas to rapidly develop core cities within the region can enhance returns to scale, thereby improving development speed and quality, and ultimately increasing economic resilience. Second, spatial agglomeration boosts economic growth until a threshold is reached, with many Chinese regions still in early agglomeration stages. Core cities, due to their higher administrative levels, often excessively draw resources from their surroundings. The urban malaise resulting from over–agglomeration can lead to urban poverty issues, outcomes that are detrimental to urban agglomerations’ ability to withstand economic shocks. Therefore, appropriate decentralization and dispersion can not only resolve their own developmental dilemmas but also promote the co–development of surrounding cities. Hinterland cities should actively embrace development diffused from core cities, fostering a balanced development gradient within the agglomeration. This approach, rather than a mere pursuit of development equalization, is more conducive to enhancing development efficiency and economic resilience. Third, considering the different industrialization stages and industrial structures of cities within urban agglomerations, it is crucial to improve the industrial division of labor among cities. Hinterland small and medium–sized cities must diverge from traditional industry reliance, hasten industrial evolution and upgrading, support core cities’ high–end industries, strengthen the regional industrial chain, and curb excessive outflows of resources and businesses. Rationalizing the industrial gradient within urban agglomerations is crucial for mitigating development disparities across China’s north and south, and between urban and rural areas.

## Supporting information

S1 Data **DATA serves as the data foundation for the part “Analyzing Trends in Dependent and Independent Variables within Chinese Urban Agglomerations” in the article.** It is used for empirical analysis and the pictorial data of Figs 12–16.
(XLSX)

## References

[1] Reggiani A, Graaff TD, Nijkamp P. Resilience: An Evolutionary Approach to Spatial Economic Systems. Networks and Spatial Economics. 2002;2(2):211–229.

[2] Simmie J, Martin R. The economic resilience of regions: towards an evolutionary approach. Cambridge Journal of Regions, Economy and Society. 2010;3(1):27–43. doi: 10.1093/cjres/rsp029

[3] Davies S. Regional resilience in the 2008-2010 downturn: comparative evidence from European countries. Cambridge Journal of Regions, Economy and Society. 2011;4(3):369–82. doi: 10.1093/cjres/rsr019

[4] Fingleton B, Garretsen H, Martin R. Recessionary shocks and regional employment: evidence on the resilience of U.K. regions. Journal of Regional Science. 2012;52(1):109–33.

[5] Pendall R, Foster KA, Cowell M. Resilience and regions: building understanding of the metaphor. Cambridge Journal of Regions, Economy and Society. 2009;3(1):71–84. doi: 10.1093/cjres/rsp028

[6] Martin R. Regional economic resilience, hysteresis and recessionary shocks. Journal of Economic Geography. 2011;12(1):1–32. doi: 10.1093/jeg/lbr019

[7] Martin R, Sunley P, Gardiner B, Tyler P. How regions react to recessions: Resilience and the role of economic structure. Regional Studies. 2016;50(4):561–85.

[8] Martin R, Gardiner B. The resilience of cities to economic shocks: A tale of four recessions (and the challenge of Brexit). Papers in Regional Science. 2019;98(4):1801–33.

[9] Brown L, Greenbaum RT. The role of industrial diversity in economic resilience: An empirical examination across 35 years. Urban Studies. 2017;54(6):1347–66.

[10] Mai X, Zhan C, Chan RCK. The nexus between (re)production of space and economic resilience: An analysis of Chinese cities. Habitat International. 2021;109.

[11] Giannakis E, Bruggeman A. Determinants of regional resilience to economic crisis: a European perspective. European Planning Studies. 2017;25(8):1394–415.

[12] Bristow G, Healy A. Innovation and regional economic resilience: an exploratory analysis. Annals of Regional Science. 2018;60:265–84.

[13] Glaeser EL, Lu M. Human–capital Externalities in China. NBER working paper. 2018;24925.

[14] Green N. Functional polycentricity: A formal definition in terms of social network analysis. Urban Studies. 2007;44(11):2077–103.

[15] Hall P. Looking forward: The city region of the mid–21st century. Regional Studies. 2009;42(6):803–17.

[16] Wei L. Analysis of regional high–quality development based on economic resilience under the new development pattern—Take 8 provinces and cites in Beijing, Tianjin, Hebei, Shanghai, Jiangsu, Zhejiang, Anhui and Guangdong as an example. Reform of Economic System. 2022;6(5):5–12.

[17] Zhang Y, Song R, Zhang K, Wang T. The Characteristics and Modes of Urban Network Evolution in the Yangtze River Delta in China from 1990 to 2017. IEEE Access. 2021;9 5531–44. doi: 10.1109/access.2020.3048948

[18] Zhang D, Kong Q, Shen M. Does polycentric spatial structure narrow the urban–rural income gap? – Evidence from six urban agglomerations in China. China Economic Review. 2023;80(1):1–20.

[19] Peng D, Wang Z, Jiang M, Kong Q. Polycentric spatial patterns and urban economic growth quality: A discussion from fintech development. Finance Research Letters. 2023;55:103932. doi: 10.1016/j.frl.2023.103932

[20] Lu H, Zhang C, Jiao L, Wei Y, Zhang Y. Analysis on the spatial–temporal evolution of urban agglomeration resilience: A case study in Chengdu–Chongqing Urban Agglomeration, China. International Journal of Disaster Risk Reduction. 2022;79. doi: INSERT_DOI_HERE

[21] Liu K, Wu Y, Wang X. Impact of spatial structure of urban agglomerations on air pollution in China. China Population, Resources and Environment. 2020;10 28–35.

[22] Zhang K, Zhang J. Polycentricity and green development efficiency of urban agglomerations: spatial distribution of urbanization based on heterogeneity. China Population, Resources and Environment. 2022(02):107–117.

[23] Wu J, Li B. Spatio-temporal evolutionary characteristics and type classification of marine economy resilience in China. Ocean & Coastal Management. 2022;217:106016. doi: 10.1016/j.ocecoaman.2021.106016

[24] Williamson JG. Regional Inequality and the Process of National Development: A Description of the Patterns. Economic Development and Cultural Change. 1965;13(4, Part 2):1–84. doi: 10.1086/450136

[25] Brülhart M, Sbergami F. Agglomeration and growth: Cross-country evidence. Journal of Urban Economics. 2009;65(1):48–63. doi: 10.1016/j.jue.2008.08.003

[26] Wang Z. Population agglomeration and regional economic development: A test for Williamson’s hypothesis under Chinese circumstance. Nanjing Journal of Social Sciences. 2018;03(03):60–9.

[27] Xiong X, Yi J, Pan Y. The impact of science and technology expenditure on urban resilience under emergencies—taking three major urban agglomerations in China as an example. Journal of Hunan University (Social Sciences). 2023;37(02):59–67.

[28] Partridge MD, Rickman DS, Ali K, Olfert MR. Do new economic geography agglomeration shadows underlie current population dynamics across the urban hierarchy?. Papers in Regional Science. 2009;88(2):445–67. doi: 10.1111/j.1435-5957.2008.00211.x

[29] Meijers EJ, Burger MJ. Spatial structure and productivity in US metropolitan areas. Environment and Planning A: Economy and Space. 2010;42(6):1383–402. doi: 10.1068/a42151

[30] Zhang D, Kong Q, Shen M. Does polycentric spatial structure narrow the urban–rural income gap? – Evidence from six urban agglomerations in China. China Economic Review. 2023;80.

[31] Wang H, Ge Q. Spatial association network of economic resilience and its influencing factors: evidence from 31 Chinese provinces. Humanit Soc Sci Commun. 2023;10(1):290. doi: 10.1057/s41599-023-01783-y 37305355 PMC10243094

[32] Teng X, Qian M, Wen C. The Effect of Polycentric Spatial Structure on Regional Economic Resilience. Journal of Technology Economics. 2022;41(06):121–30.

[33] Li S, Lee S, Liu X. Economic performance of polycentric spatial structure based on city-region scale. China Population, Resources and Environment. 2021;31(11):123–33.

[34] Yin H, Hong T, Ma T. Urban network externalities, agglomeration economies and urban economic growth. Cities. 2020;107:102882. doi: 10.1016/j.cities.2020.102882

[35] Huang J, Li Q, Zhong P. Spatial differences and dynamic evolution of economic resilience in China’s eight major urban agglomerations. Statistics & Decision. 2022;38(17):91–6. doi: 10.13546/j.cnki.tjyjc.2022.17.018

[36] Brown L, Greenbaum RT. The role of industrial diversity in economic resilience: An empirical examination across 35 years. Urban Studies. 2016;54(6):1347–66. doi: 10.1177/0042098015624870

[37] Chen J, Li X, Zhu Y. Shock absorber and shock diffuser: the multiple roles of industrial diversity in shaping regional economic resilience after the Great Recession. Ann Reg Sci. 2023;72(3):1015–45. doi: 10.1007/s00168-023-01233-2

[38] Zhang M, Wu Q, Li W. Industrial structure transformation, total factor productivity and urban economic resilience. Journal of Zhengzhou University (Philosophy and Social Sciences Edition). 2021;54(06):51–7.

[39] Meijers E, Hoogerbrugge M, Cardoso R. Beyond Polycentricity: Does Stronger Integration Between Cities in Polycentric Urban Regions Improve Performance? Tijd voor Econ & Soc Geog. 2017;109(1):1–21. doi: 10.1111/tesg.12292

[40] Li Y, Phelps NA. Knowledge polycentricity and the evolving Yangtze River Delta megalopolis. Regional Studies. 2016;51(7):1035–47. doi: 10.1080/00343404.2016.1240868

[41] Xu X, Lei Z, Dou Y, Liu S. Research on gap of balanced development between the north and the south of China——Analysis based on “China balanced development index”. China’s Industrial Economics. 2021;2(5):5–22.

[42] Mao J, Wang D, Bai C. Intergovernmental transfers for ethnic regions, public spending difference and economic development gap. Economic Research Journal. 2011;46(S2):75–87.

[43] Zhang Z, Wu D. Industrial spatial agglomeration, factor mobility and regional balanced development: From the perspective of urban economic development gap in the Yangtze River economic zone. Reform of Economic System. 2019;4:42–8.

[44] Zhang M, Wu Q, Li W. Industrial structure change, total factor productivity and urban economic resilience. Journal of Zhengzhou University (Philosophy and Social Sciences Edition). 2021;54(06):51–7.

[45] Zhang Z, Li Z, Hu X. Industrial agglomeration, spatial spillover, and regional economic resilience of urban agglomeration. East China Economic Management. 2021;35(08):59–68. doi: 10.19629/j.cnki.34-1014/f.210307001

[46] Parr J. The Polycentric Urban Region: A Closer Inspection. Regional Studies. 2004;38(3):231–40. doi: 10.1080/003434042000211114

[47] Meijers EJ, Burger MJ, Hoogerbrugge MM. Borrowing size in networks of cities: City size, network connectivity and metropolitan functions in Europe. Papers in Regional Science. 2016;95(1):181–99. doi: 10.1111/pirs.12181

[48] Lu M, Li P, Zhong H. The new era of development and balance: Spatial political economics of new China’s regional economy for 70 years. Journal of Management World. 2019;35(10):11–23. doi: 10.19744/j.cnki.11-1235/f.2019.0128

